# Floral Nectar Guide Patterns Discourage Nectar Robbing by Bumble Bees

**DOI:** 10.1371/journal.pone.0055914

**Published:** 2013-02-13

**Authors:** Anne S. Leonard, Joshua Brent, Daniel R. Papaj, Anna Dornhaus

**Affiliations:** 1 Department of Biology, University of Nevada, Reno, Reno, Nevada, United States of America; 2 Department of Ecology and Evolutionary Biology, University of Arizona, Tucson, Arizona, United States of America; Emory University, United States of America

## Abstract

Floral displays are under selection to both attract pollinators and deter antagonists. Here we show that a common floral trait, a nectar guide pattern, alters the behavior of bees that can act opportunistically as both pollinators and as antagonists. Generally, bees access nectar via the floral limb, transporting pollen through contact with the plant’s reproductive structures; however bees sometimes extract nectar from a hole in the side of the flower that they or other floral visitors create. This behavior is called “nectar robbing” because bees may acquire the nectar without transporting pollen. We asked whether the presence of a symmetric floral nectar guide pattern on artificial flowers affected bumble bees’ (*Bombus impatiens*) propensity to rob or access nectar “legitimately.” We discovered that nectar guides made legitimate visits more efficient for bees than robbing, and increased the relative frequency of legitimate visits, compared to flowers lacking nectar guides. This study is the first to show that beyond speeding nectar discovery, a nectar guide pattern can influence bees’ flower handling in a way that could benefit the plant.

## Introduction

Floral patterns have intrigued pollination biologists for centuries. From the classic observations of Sprengel [1] to the discovery of UV nectar guides [2], [3] to modern work on the genetic architecture of floral pigmentation [4], researchers have largely focused on understanding how floral patterns promote pollinator visitation. For example, pollinator preferences for specific pattern components are well-studied [5]–[7], and pollinators can clearly use these patterns to distinguish between flower types that differ in reward value [8], [9]. Yet although floral patterns are very common among animal-pollinated plants [10], surprisingly, we know little about how they affect the behavior of floral visitors whose behavior reduces plant fitness. Here we ask whether nectar guides influence the behavior of floral visitors that can act flexibly as either mutualists or antagonists, bumble bees that rob nectar opportunistically.

**Figure 1:**
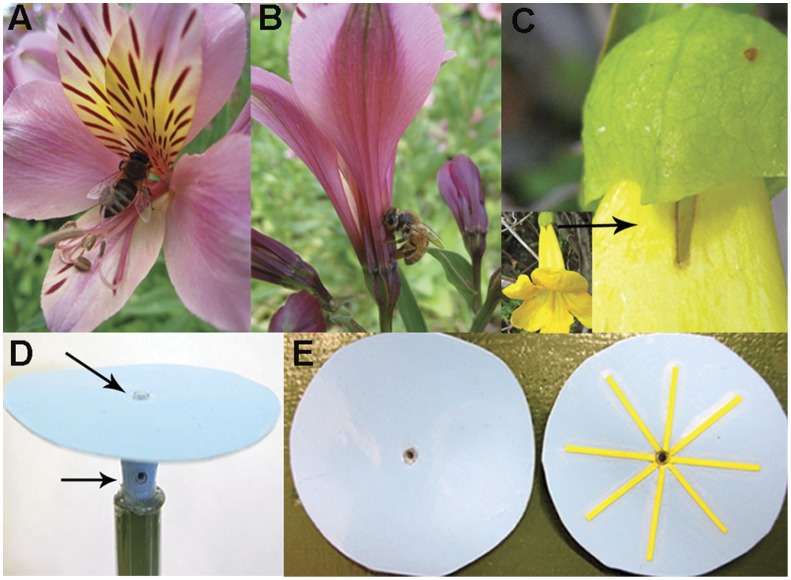
Robbers may access nectar via holes that they or previous visitors create. (A) Honey bee (*Apis mellifera*) visiting *Alstroemeria*, a flower with a nectar guide pattern. (B) *A. mellifera* robbing *Alstroemeria*. (C) Access hole left by primary robber on *Tecoma sp.* (D) Experimental flowers had two sucrose wells, located on the top and tube. (E) Floral tops had either a yellow nectar guide, or were plain. Photographs: A.S. Leonard.

Nectar robbing [11] occurs when, rather than accessing nectar “legitimately” from the plant perspective (Fig. 1a), a visitor instead uses a hole in the base of the flower (Fig. 1b). A visitor may engage in primary robbing by creating the hole, or secondary robbing by using a hole left by a primary robber (Fig. 1c; [12]). Certain bee species can adopt a flexible flower handling strategy: for example, some species are capable of both primary and secondary robbing [13], and may both rob and visit legitimately [14], [15], even during visits to the same flower [16]–[18].

The interests of partners in a mutualism may not always coincide [19], and nectar robbing appears to place the reproductive interests of the plant and floral visitor in potential conflict. Robbing may increase bees’ rate of nectar collection [20], but a robber that bypasses the anther and stigma to gain nectar without transferring pollen, in the 18^th^ century assessment of Sprengel “commits an outrage against a flower” [1]. From the plant’s perspective, this foraging strategy represents a loss of nectar that could have been used to reward a subsequent pollinator [21], [22]; it can also, of course, represent a lost pollination opportunity.

Despite bees’ widely observed flexibility in flower handling [23]–[25], the floral traits that elicit robbing are not well understood [11], [26], [27]. For example, it is unknown whether floral signals can influence a floral visitor’s decision to rob. Yet, patterns on flowers can function as “nectar guides” [28](Fig. 1a), directing pollinators towards the nectary and reproductive structures of the plant. Nectar guides reduce the time bees spend on the flower [29], [30], presumably leading to a faster rate of nectar collection. Nectar guide patterns often match bees’ color and shape preferences [9], so they can also promote pollen transfer for the plant by attracting bees to the flower. Although research on nectar guides dates to the early days of pollination biology [31], no previous study has explored whether these signals affect a bee’s propensity to legitimately pollinate. Therefore, we asked: does a nectar guide influence a bee’s handling strategy in a way that benefits the plant?

Our experiment used artificial flowers whose nectar wells could be accessed both by extracting nectar from the side of the floral tube (“robbing”) and by landing on the surface of the floral top (“legitimate visits”). This design allowed us to distinguish two possible effects of patterns on flower handling. First, nectar guides might decrease robbing behavior. Secondly, nectar guides might increase legitimate visits. Since flowers offered rewards to legitimate visitors as well as to robbers, these possibilities were not mutually exclusive.

Comparing the handling times associated with each type of visit allowed us to also assess the costs and benefits of nectar guides to plants and bees. For example, a floral pattern might exploit bees’ innate landing preferences for certain aspects of color and shape [7], [28] (but see [32]); a floral pattern might also speed nectar discovery [29], [30]. Either effect might increase legitimate visits to patterned flowers, but with different consequences for bees’ foraging performance. If nectar guides make legitimate visits faster than robbing, this would promote both pollen transfer and nectar collection rate. If, however, robbing is always faster, then nectar guides that promote legitimate visits would benefit the plant but conceivably reduce bees’ foraging performance.

## Methods

We trained 77 *Bombus impatiens* foragers (worker caste) to visit a horizontal floral array individually in an experimental chamber connected to the colony via gated mesh tubing (L x W x H: 3.05 m x 1.92 m x 1.72 m). The colony was provided with pollen *ad libitum,* and housed in a plastic box (L x W x H: 22.0 cm x 24.0 cm x 12.0 cm). Bees were fitted on the thorax with numbered tags (E.H. Thorne Ltd., Wragby, Lincolnshire, UK) for identification.

The horizontal floral array consisted of a Styrofoam base (60 cm x 60 cm) painted green (DecoArt acrylic paint, “Avocado” #DA052) and located in the center of the experimental chamber, at a height of 50 cm above the ground. The chamber was illuminated by fluorescent lighting (Sylvania Cool White 34 Watt bulbs, # F40CW1SS, 60 Hz, 560 lux measured at center of array).

### Training

To induce bees to visit the experimental chamber, we provided them with overnight access to a single multi-port glass feeder filled with 35 ml of 30% (wt/wt) sucrose solution located at the site of the foraging array. Twice a week, bees underwent an additional training procedure. In order to induce bees to land on artificial flowers, for 4–6 hours on these days, bees were allowed free access to a horizontal array that offered six blue training flowers, arranged in the same position as foraging trial flowers (10.0 cm apart, in a 3×2 grid). Flowers bases were constructed from a 1 ml transparent pipette tip (L: 7.0 cm), painted internally with the same green as the Styrofoam board. Training flowers’ base contained a cotton wick, which was moistened with 1 ml of 30% sucrose solution and accessible to bees through a small hole in the floral top. In order to prevent bees from learning to feed from the center of each training top, we varied the position of the wick across flowers (near center vs. near edge). While this pre-training to land on the tops of flowers may have biased bees to land on the tops of experimental flowers, we observed that they in fact robbed experimental flowers frequently (see Results). The sucrose concentrations used in this experiment (30%; 50%) are within the range bees might naturally encounter [33], [34]; we used a higher concentration in foraging trials to encourage bees to visit experimental flowers.

### Foraging Trials

Six flowers were present on the floral array during foraging trips (Fig. 1d). Each floral top had one centrally located well, which offered 3.0 µl of 50% sucrose through a 2.5 mm diameter opening. Floral tops were 5.0 cm diameter circles, constructed from water resistant paper that was painted blue (DecoArt Baby Blue #DA042) and laminated. Before lamination, we added a yellow star-shaped pattern to the tops of certain flowers, by placing four strips (L: 4.0 cm W: 1.0 mm) of pressure-sensitive labeling tape (VWR International, Radnor, PA) in a radial arrangement (Fig. 1e). We assumed that lamination would mask any volatiles released from the tape, rendering the nectar guide a primarily visual stimulus.

The floral tube was constructed from a 2.0 cm section of a transparent pipette tip, painted (internally) the same blue as the flower top. The tube offered a 2.5 mm diameter sucrose well, located 5.0 mm from the top, filled with 3.0 µl of 50% sucrose. Each sucrose well was constructed from the terminal 5.0 mm section of a 200 µl pipette tip, sealed at the tip with hot glue. All flowers were cleaned with 30% ethanol between foraging trials to remove any scent marks deposited by foragers; lamination of tops and internal painting of plastic components ensured that this cleaning did not affect paint. Between foraging trials, we used a glass capillary tube to confirm that bees in fact emptied the 3 µl of sucrose from wells that we had observed them probing.

We randomly assigned bees to one of two foraging conditions: nectar guides present (all tops had nectar guides, N = 39) or nectar guides absent (“plain”: all tops lacked nectar guides, N = 38). Each bee experienced only a single foraging trip in our setup, and was the only bee in the experimental chamber during that foraging trip. The experiment was run from 6/27/10 to 8/11/10; to control for potential effects of colony age on foraging behavior, we generally tested bees assigned to each condition each day (all days except for 3). During a trial, we videotaped the bee foraging on the array for 10 minutes (30 frames/s; Sony DVM-60PR Mini DV cassettes), or until it was away from the array for more than 3 minutes. We scored a floral landing as “legitimate” if the bee landed upon the floral top, and “robbing” if the bee landed on the floral tube after approaching it from the underside. We then used iMovie 8.0.6 (Apple Computer Inc., California, USA) to measure the frame-by-frame details of nectar discovery on the first and last flowers fed from via both legitimate and robbing landings. We defined nectar discovery time as the interval between the bee landing on (first touching) the flower and feeding from the sucrose well (inserting its proboscis).

Sample sizes for certain comparisons were reduced if bees landed 0–1 times on floral tops (N = 10), or had an unrecorded landing (N = 1). When necessary, we transformed data to meet the assumptions of normality and equal variance; when this was not possible, we used non-parametric analyses [35], [36].

## Results

### The Effect of Nectar Guides on Nectar Discovery Speed

**Figure 2:**
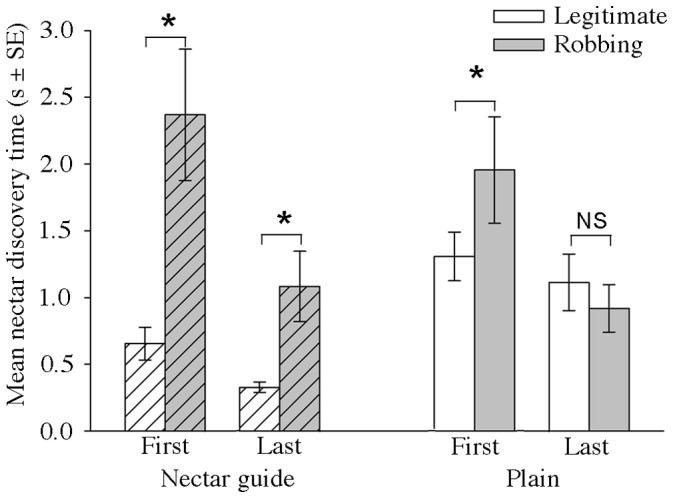
Discovery times on plain and patterned flowers at the beginning and end of foraging trips. On both the first and last flower of the trip, bees visiting flowers legitimately discovered nectar more quickly when flowers were patterned (diagonal hatched lines). When flowers were patterned, legitimate foraging was faster than robbing; when flowers were plain, discovery times for both handling strategies were ultimately similar. See Table 1 for analysis; asterisks indicate results of post-hoc paired t-tests.

**Figure 3:**
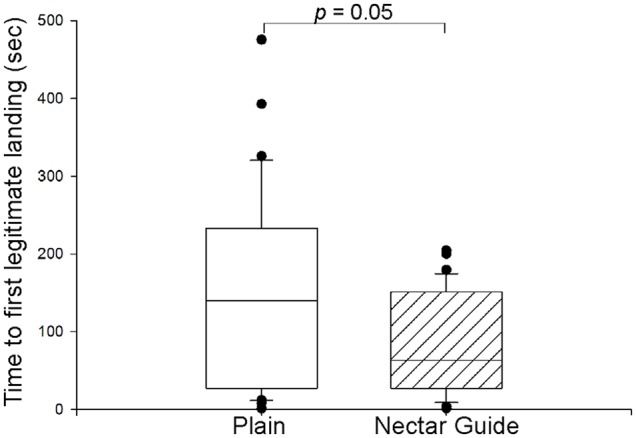
Floral patterns and time to first legitimate landing. When flowers had nectar guides (diagonal hatched lines), bees landed legitimately sooner after the start of the trial than when flowers were plain. Horizontal lines represent medians; boundaries represent 25^th^ and 75^th^ percentiles; whiskers represent 10^th^ and 90^th^ percentiles. See text for analysis.

We used the General Linear Model procedure in SPSS (v.21, IBM Corporation) to assess how the presence of a floral nectar guide pattern, floral order within the trip, and flower handling strategy affected nectar discovery time. First, to confirm that the star-shaped patterns functioned as nectar guides, we compared how quickly bees discovered nectar on plain vs. patterned tops (Fig. 2, Table 1). Because we expected that bees’ nectar discovery time would decrease over the course of a foraging trip [30], we made this comparison for bees on both their first and their last visit of the foraging trip, separately for legitimate and robbing visits. On their first visit, bees discovered nectar on average twice as quickly when flowers had patterns (*p* = 0.002). Although nectar discovery time decreased with experience (Flower order: *p*<0.001), the patterns’ effect on discovery time persisted (Pattern x Flower order: *p*>0.05): even on the last flower visited during the foraging trip, discovery time for legitimate visits to patterned flowers occurred on average three times as quickly as on plain flowers. We also observed a trend for bees to made their first legitimate landing sooner after the start of the trial when flowers were patterned (Fig. 3; medians: plain: 139.9 s; patterned: 63.3 s; Mann-Whitney U = 414.5, *p* = 0.05), indicating that search time for flowers (in addition to discovery of the nectar once on the flower) was also reduced when flowers were patterned. Thus, we conclude that the patterns functioned effectively as nectar guides throughout the foraging trip, initially attracting bees to floral tops and speeding nectar discovery after landing.

**Table 1:** Summary of General Linear Model.

Factor	*F*_1,64_	*P*	
Pattern *(plain vs. nectar guide)*	10.824	**0.002**	
Flower order *(first vs. last)*	18.858	**<0.001**	
Landing type *(legitimate vs. robbing)*	8.396	**0.005**	
Landing type x Pattern	37.441	**<0.001**	
Landing type x Flower order	0.987	0.324	
Flower order x Pattern	0.703	0.405	
Landing type x Flower order x Pattern	1.673	0.200	

Analysis of the effects of flower pattern, flower order within the foraging trip, and landing type on nectar discovery time.

### Efficiency of Robbing vs. Legitimate Foraging

The relative handling time benefit of robbing depended upon whether floral tops were patterned or plain (Fig. 2; Table 1). When tops were plain, accessing nectar legitimately took a similar amount of time as robbing; when tops had nectar guides, foraging legitimately was significantly faster than robbing, occurring approximately 3.5 times as quickly on average (Pattern x Landing type *p*<0.001). As noted above, the discovery time associated with robbing itself decreased with experience, to a similar extent as legitimate discovery time (Flower order x Landing type *p*>0.05): the overall model suggests that the relative difference in speed of robbing versus legitimate visits thus did not change with experience. To explore in more detail the relationship between handling strategy, discovery time, and experience, we performed four post-hoc paired t-tests, with a Bonferroni-adjusted á = 0.0125 [36] comparing the speed of robbing vs. legitimate nectar discovery for bees on their first and last plain and patterned flowers. This analysis confirmed that legitimate foraging was faster for bees on patterned flowers on both the first (t_36_ = −4.521, *p*<0.001) and last (t_36_ = 5.187, *p*<0.001) flowers. It showed that while legitimate foraging was faster than robbing for bees on their first plain flower (t_29_ = 3.011, *p* = 0.005), this difference had disappeared by the last flower (t_30_ = 1.065, *p* = 0.295).

### Did Bees Land Legitimately More and/or Rob Less When Flowers had Nectar Guides?

**Figure 4:**
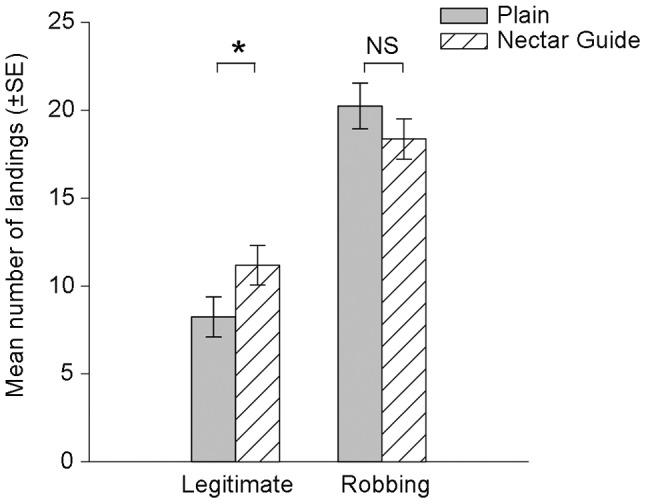
Floral patterns and landing type. When flowers had nectar guides (diagonal hatched lines), bees made a greater number of legitimate landings on the floral top than when they were plain. See text for analysis.

Bees made a greater proportion of all landings on floral tops when flowers had nectar guides (Mean ± SE: Nectar guides: 0.37±0.029; Plain: 0.28±0.030; t_75_ = 2.157, *p = *0.034). Bees landed significantly more often on tops when flowers had nectar guides than when they were plain (Fig. 4; Mann-Whitney U = 540.5, *p = *0.041) and consequently collected more sucrose legitimately (median collected: nectar guides: 18 µl; plain: 15 µl; Mann-Whitney U = 538.5, *p = *0.021).

Bees tended to rob more frequently than they landed legitimately. We found no significant difference between the number of robbing visits when flowers were plain or patterned (Fig. 4; t_75_ = −1.086, *p* = 0.281), and bees in both treatments collected a similar volume of sucrose via robbing (median collected: nectar guides: 18 µl; plain: 18 µl; Mann-Whitney U = 632.5, *p = *0.158).

## Discussion

Nectar guides are common floral traits that both attract pollinators [7], [37], [38] and speed nectar discovery [29], [30]. Our experiment suggests a new functional perspective on these long-studied floral signals: nectar guides make the handling strategy that benefits the plant more efficient for the pollinator. Patterns induced bees to extract nectar legitimately more frequently, thereby potentially transferring more pollen between flowers. Additionally, nectar guides decreased the time it took bees to locate sucrose legitimately, relative both to plain flowers and to robbing. Although nectar guides did not completely defend flowers against robbing, bees’ increased tendency to forage legitimately in their presence yielded a greater volume of sucrose, collected more quickly. In this scenario, the benefits of nectar guides are thus potentially shared by both plant and pollinator.

### Proximate Causes of Nectar Robbing

The sensory and cognitive bases of nectar robbing are surprisingly unexplored [11]. By observing the responses of relatively flower-naïve bees in a controlled setting, our experiment reveals some of the proximate factors that contribute to opportunistic robbing. This experimental approach is crucial for understanding how flower handling strategies emerge from a bee’s response to the sensory architecture of the flower.

Clearly, both skill at robbing and propensity to rob can depend upon experience. For example, we found that bees’ robbing speed increased over the course of a foraging trip, a result consistent with earlier observations that robbing effectiveness improves with time [39]. Interestingly, the relative time benefit of foraging legitimately vs. robbing did not change over the course of a trip, demonstrating that both relatively naïve and experienced foragers face similar relative time costs associated with each handling strategy.

It is worth noting that nectar robbing can also be socially facilitated among bumble bees: Leadbeater and Chittka [40] demonstrated that *B. terrestris* foragers are faster to rob if they have previously observed conspecifics doing so; bees are also more likely to become primary robbers if they have previously robbed secondarily. Since nectar robbers remain outside the corolla whereas petals may hide legitimate visitors from view, it is possible that social facilitation might even occur more effectively for nectar robbing. To understand our results in a more complex foraging scenario, an obvious next step would be to simultaneously compare the relative influence of nectar guides and conspecifics on the development of nectar robbing behavior.

### Floral Signaling as a form of Resistance to Nectar Robbing?

Interactions with multiple selective agents have shaped the evolution of floral phenotypes [41]. For example, nectar constituents are involved both in rewarding pollinators and in defending the plant against herbivores [42]–[44]. Likewise, floral pigments that produce patterns attractive to pollinators may also deter florivores [45]. Our experiment suggests an additional but complimentary perspective: in addition to attracting pollinators, visual signals may be a form of resistance to nectar robbing [11], in that they reduce consumer damage by increasing the odds that a bee will forage legitimately.

Most commonly, studies of floral resistance to nectar robbing have focused on structural or physiological traits (e.g. thickening of floral tissues, bracts, or calyces; clustering into inflorescences; secondary compounds in nectar [39], [44]). To our knowledge, our experiment is the first to explore whether a floral signal affects robbing behavior. This is noteworthy, because signals have the potential to deceive or manipulate the sensory system of receivers [46], [47]. For example, floral patterns may exploit bees’ landing preferences for particular colors and shapes, such as those associated with pollen or nest entrances [37], [48]–[50]. In these cases, the floral signal has the potential to promote pollen transfer for the plant at the expense of a pollinator’s foraging efficiency [30], [51].

The hypothesis that bees rob in order to collect nectar more quickly dates to Darwin [52], and has been supported by a number of subsequent investigations [16], [20]. The relative time costs of robbing vs. legitimate foraging will clearly depend upon the morphology of both flower and bee (e.g. floral tube vs. proboscis length, size of floral diameter vs. bee diameter [39], [53]). Our results thus suggest several lines of research to further explore the relationship between floral structure, signaling, and nectar robbing. For example, variation in the relative surface areas of floral tubes and tops might alter the relationship between nectar guides and nectar robbing, or influence the size or color that a nectar guide needs to be for maximum effectiveness. It would also be interesting to assess our findings across a diversity of patterns and colonies.

The stability of a mutualism depends upon features that make cooperation pay off more than defection for both partners [14]. Cheating is a strategy that may be available to both plant and pollinator: bees can acquire nectar without transferring pollen, and plants can induce bees to visit regardless of reward presence or time cost [54]. Indeed, floral patterns have recently been highlighted as potentially having evolved to exploit ancestral insect species’ preexisting sensory biases [37], [46], [47]. On an evolutionary timescale, such sensory exploitation might allow plants to increase visitation rates while limiting rewards. However, our findings demonstrate that rather than driving the evolution of floral deception, floral patterns may play a role in a process of evolutionary “negotiation” between plant and pollinator, maintaining mutual benefits: when it comes to handling strategy, nectar guides make cooperation profitable for bees.
